# 

**Published:** 1894-03-17

**Authors:** 


### CONTENTS.

Leaderless Medicine - - - - - - - - - - 429
Around the Hospitals - - - - - - - - - - 430
Temperature and Anæsthesia. By Sir BENJAMIN WARD RICHARDSON, M.D., F.R.S. - - - - - - - - 431
Art and Disease. VIII.—In the National Gallery - - - 432
Medical Progress and Hospital Clinics—
Empyema of the Antrum (*continued*) - - - - - - 437
Edinburgh Royal Infirmary—Catarrhal Jaundice and its Treatment - - - - - - - - - - 438
MEDICAL PROGRESS.—Diseases of Children - - - - - 439
Asthma - - - - - - - - - - - 442
NEW DRUGS AND PREPARATIONS - - - - - - - 442
NEW APPLIANCES AND THINGS MEDICAL - - - - - 444
Modern Aspects of the Treatment of Graves' Disease - 433
The Field of Science - - - - - - - - - - 434
Annotations.—Headaches—Rest the Recuperator—Metropolitan Hospital Saturday Fund - - - - - - - - 435
Notes and News - - - - - - - - - - - 436
Editor's Letter-Box.—The Red Cross Ambulance - - - 444
The Institutional Workshop—
WITHIN THE HOSPITALS—Wrexham Infirmary - - - - 445
Aberdeen and its Institutions - - - - - - 445
PRACTICAL DEPARTMENTS - - - - - - - - 446
HOSPITAL FESTIVALS, MEETINGS, &c. - - - - - - 448
Scraps and Gleanings - - - - - - - - - 448
The Book World of Medicine and Science - - - - 449

EXTRA SUPPLEMENT.—THE NURSING MIRROR.

News from the Nursing World.—Subjects for Sermons—Bold through Ignorance—Dignity in Danger—Trained Nursing in Workhouses—Good Work at Alfreton—"In the Nurse's Absence"—The Visiting Nurse—District Nurses for Nottingham—Wanted, a Cottage Hospital—A Prosperous Society—Gaiety at Glasgow—Mixed Motives—Pleasant Environs—Australian Health Society—Short Items - - ccxxxi
On General Nursing. III.—Scarlet Fever - - - - - ccxxxiii
Nursing in Africa.—A Night in a South African Asylum - ccxxxiv
The Training of Male Nurses - - - - - - - ccxxxv
Notes and Queries - - - - - - - - - - ccxxxv
Women's Work.—Typewriting - - - - - - - ccxxxvi
Women's Volunteer Medical Staff Corps - - - - ccxxxvi
Novelties for Nurses - - - - - - - - - ccxxxvi
Nursing in the United States - - - - - - - ccxxxvii
For Reading to the Sick - - - - - - - - ccxxxvii
Australian Notes—Appointments - - - - - - ccxxxviii
The Muses' Looking Glass.—"A Daisy Under its Wing" ccxxxix
Minor Appointments - - - - - - - - - - ccxxxix
Everybody's Opinion - - - - - - - - - - ccxl

